# Supplementary material for: Patterns of Intron Gain and Loss in Fungi
Source: PLoS Biol. 2004 Nov 30;2(12):e422. doi: 10.1371/journal.pbio.0020422 (PMC532390; doi:10.1371/journal.pbio.0020422)
Supplement: Table S1 — Also available at http://genes.mit.edu/NielsenEtAl/. (4.3 MB ZIP). [file pbio.0020422.st001.zip › NielsenEtAl/html/607.html]

AN1964.1.NCU08502.1.MG03236.1.FG00634.1


```
 CLUSTAL W (1.82) Multiple Sequence Alignments - Introns Inserted


Sequence 1: NCU08502.1	239 aa
Sequence 2: FG00634.1	237 aa
Sequence 3: MG03236.1	239 aa
Sequence 4: AN1964.1	237 aa
Alignment Length: 239 aa
Number Identitical Residues: 155 aa
Alignment Score (without introns) 7089


MG03236.1 	MK~FNISNPSTGAQKLVEVEDERKLRFFME~KR0MGAE~VSAEPLGDEWKGYILKITGGN
NCU08502.1	MK0LNISYPANGSQKLIEVEDERKLRHFIE2KR0MGAE~VPADPLGDEWKGYILRITGGN
FG00634.1 	--~MNISYPANGSQKLIDIEDERKVRVFMD~KR0MGAE~VPGDSVGDEFKGYIFRITGGN
AN1964.1  	MK~LNISYPANGSQKIVEIDDERKLRPFME~KR~MGTE0VPGDSLGDEFKGYLFKITGGN
          	 . :*** *:.*:**:::::****:* *:: ** **:* *..:.:***:***:::*****

MG03236.1 	DKQG~FPMKQGV~LLPNRTRLLLSDGHSCYRARRTGERKRKSVRGCITGMDLSVLAVSIA
NCU08502.1	DKQG~FPMKQGV~IAPNRVRLLLSDGHSCYRARRTGERKRKSVRGCIVGSDLSVLALAIV
FG00634.1 	DKQG~FPMKQGV~MHPTRVRLLLSEGHSCYRPRRTGERKRKSVRGCIVAMDLSVLALSIV
AN1964.1  	DKQG1FPMKQGV1LLPTRTRLLLADGHSCYRPRRTGERKRKSVRGAITGQDLAVLALSIV
          	**** ******* : *.*.****::******.*************.*.. **:***::*.

MG03236.1 	KRGEQDIPGLTDVVHPKRLGPKRATKIRKFFDLSKEDD0VRKYVIRREVQPKGEGKKPYT
NCU08502.1	KQGEQDIPGLTDVVHPKRLGPKRATKIRRFFGLSKDDD0VRKYVIRREVQPKGEGKKPYT
FG00634.1 	KQGDADIPGLTDVVHPKRLGPKRATKIRKFFGLTKDDD0VRKYVIRREVQPKGEGKSPYT
AN1964.1  	KQGEGELPGLTDTVVPKRLGPKRATKIRNFFGLDKKDD~VRKFVIRRTVTK--EGKKDYT
          	*:*: ::*****.* *************.**.* *.** ***:**** *    ***. **

MG03236.1 	KAPKIQRLVTPQRLQHKRHRIALKKRQSEKVKDAA0NEYAQILAKRVAEAKATKADARKR
NCU08502.1	KAPKIQRLVTPQRLQHKRHRLALKRRQAEKVKEEA0SEYAQILAKRIAEAKVQKADARKR
FG00634.1 	KAPRIQRLVTPQRLQHKRHRAALKRRQAEKVKDEA0NEYAQVLAKRIAEAKVNKADARKR
AN1964.1  	KAPKIQRLVTPQRLQRKRHRIALKRRRAEAAREAA2NDYAKLLASRVHEEKAKKDELRKR
          	***:***********:**** ***:*::* .:: * .:**::**.*: * *. * : ***

MG03236.1 	RASSMRK
NCU08502.1	RASSMRK
FG00634.1 	RASSMRK
AN1964.1  	RASSMRK
          	*******
```
